# Calibration of the global physical activity questionnaire to Accelerometry measured physical activity and sedentary behavior

**DOI:** 10.1186/s12889-018-5310-3

**Published:** 2018-03-27

**Authors:** Kristen M. Metcalf, Barbara I. Baquero, Mayra L. Coronado Garcia, Shelby L. Francis, Kathleen F. Janz, Helena H. Laroche, Daniel K. Sewell

**Affiliations:** 1 University of Iowa, 102 Field House, Iowa City, IA 52242 USA; 2 University of Iowa, N418 CPHB, Iowa City, IA 52242 USA; 3 University of Iowa, N300 CPHB, Iowa City, IA 52242 USA; 4 University of Iowa, SW34 GH, Iowa City, IA 52242 USA; 5 University of Iowa, E130 Field House, Iowa City, IA 52242 USA; 6 University of Iowa, 280B MRF, Iowa City, IA 52242 USA; 7 University of Iowa, N338 CPHB, Iowa City, IA 52242 USA

**Keywords:** Accelerometry, Calibration, Physical activity, Sedentary behavior, Self-report, Measurement, Public health

## Abstract

**Background:**

Self-report questionnaires are a valuable method of physical activity measurement in public health research; however, accuracy is often lacking. The purpose of this study is to improve the validity of the Global Physical Activity Questionnaire by calibrating it to 7 days of accelerometer measured physical activity and sedentary behavior.

**Methods:**

Participants (*n* = 108) wore an ActiGraph GT9X Link on their non-dominant wrist for 7 days. Following the accelerometer wear period, participants completed a telephone Global Physical Activity Questionnaire with a research assistant. Data were split into training and testing samples, and multivariable linear regression models built using functions of the GPAQ self-report data to predict ActiGraph measured physical activity and sedentary behavior. Models were evaluated with the testing sample and an independent validation sample (*n* = 120) using Mean Squared Prediction Errors.

**Results:**

The prediction models utilized sedentary behavior, and moderate- and vigorous-intensity physical activity self-reported scores from the questionnaire, and participant age. Transformations of each variable, as well as break point analysis were considered. Prediction errors were reduced by 77.7–80.6% for sedentary behavior and 61.3–98.6% for physical activity by using the multivariable linear regression models over raw questionnaire scores.

**Conclusions:**

This research demonstrates the utility of calibrating self-report questionnaire data to objective measures to improve estimates of physical activity and sedentary behavior. It provides an understanding of the divide between objective and subjective measures, and provides a means to utilize the two methods as a unified measure.

## Background

Physical activity (PA) is important as an exposure and outcome variable in public health research. Although objective measurement of PA using accelerometry is widely used in public health research, its costs, complexities, and participant burden preclude its use in all studies. As such, self-report measures of PA, including questionnaires, remain a valuable method of PA measurement that can provide unique and valuable information about domains in addition to estimates of volume of PA. Furthermore, self-report measures of PA allow for comparison of longitudinal data where objective measures were not utilized in all measurement periods. When compared to accelerometry, the most commonly used self-report measures of PA, e.g., the International Physical Activity Questionnaire, the Global Physical Activity Questionnaire (GPAQ), and the Seven-Day Physical Activity Recall show low to moderate validity of 0.17–0.41 [1–4]. Clearly, there is a need to improve the accuracy of PA self reports. For these reasons, many public health research studies utilize both objective and self-report measures of PA, such as the National Health and Nutrition Examination Survey, the Women’s Health Study, and the Iowa Bone Development Study [5–7].

The GPAQ was developed by the World Health Organization (WHO) for PA surveillance, including the assessment of PA trends over time [8]. The GPAQ is available in nine languages, and has been utilized as a part of the WHO STEPwise Approach to Non-Communicable Chronic Disease Risk Factor Surveillance in over 100 countries [9]. The GPAQ collects information about PA in three domains: work, travel, and recreation, as well as average time per day spent in sedentary behavior (i.e., sitting and reclining) [10]. It is scored in minutes per day (min/d) to provide meaningful behavioral units, rather than a unit-less questionnaire score that is difficult to interpret, e.g., the Godin-Shephard Leisure-Time Exercise Questionnaire [11]. The first version of the GPAQ was validated in multiple populations, including adults from the Bangladesh, Brazil, China, Ethiopia, India, Indonesia, Japan, Portugal, South Africa, the United States, and Vietnam. The GPAQ had low to moderate validity for total PA when compared to pedometers (*r* = 0.31–0.39) [8, 12, 13] and accelerometers (*r* = 0.20–0.34) [14]. Validity appeared lower for vigorous-intensity PA (*r* = 0.23–0.26). However, the first version of the GPAQ showed good test-retest reliability (*r* = 0.67–0.81) [8, 12]. This suggests that while the GPAQ may not be appropriate for cross-sectional studies due to its low to moderate validity, it may be a good tool for assessing changes in PA over time.

The GPAQ was revised to improve wording and shorten its length. Herrmann and colleagues examined the second version’s validity by comparing it to PA measured using the ActiGraph accelerometer and the Yamax pedometer. In adults, the GPAQ had a low level of agreement with the ActiGraph for moderate- and vigorous-intensity PA (MVPA) (*r* = 0.26) and a moderate level of agreement with the Yamax pedometer for steps/day (*r* = 0.39). Associations were negative for sedentary behavior, with a non-significant level of agreement between the GPAQ and ActiGraph (*r* = − 0.12). However, once again, the GPAQ showed good short-term reliability, with coefficients ranging from *r* = 0.83–0.96 [2]. Cleland and colleagues assessed the validity of the second version of the GPAQ in adults living in the United Kingdom, and reported a moderate level of agreement between the GPAQ and ActiGraph measured PA (*r* = 0.48). The GPAQ had a moderate, but non-significant agreement with the ActiGraph for change over time in MVPA (*r* = 0.52) and a non-significant negative agreement of change over time in sedentary behavior (*r* = − 0.02). These researchers also found a negative bias with the GPAQ, indicating that individuals who were more active, were more likely to over-report PA [3].

Recently Saint-Maurice and colleagues demonstrated a calibration strategy to improve interpretability of the Physical Activity Questionnaire for Children and Physical Activity Questionnaire for Adolescents. They simultaneously measured PA in children and adolescents with questionnaires and accelerometers, then used multivariable linear regression to calibrate the relationship between questionnaire scores and ActiGraph measured MVPA [15]. Welk and colleagues developed a similar method to adjust for error in the 24-Hour Physical Activity Recall (PAR) in adults, by simultaneously measuring PA with the 24-Hour PAR and the SenseWear Armband Mini [16]. The 24-h PAR queries contextual PA information like that of the GPAQ (e.g., domain), however it takes on average 20 min to complete, and inquires about the previous 24 h. These studies showed the feasibility of calibrating self-reported PA data to objectively measured PA, and the ability to improve their accuracy and usability. The current study used a similar strategy to provide a unique way to calibrate and compare past and present self-reported PA measures with objective measures, which are the current standard for PA measurement. It also allowed for better understanding of the direction and level of disconnect between self-reported and objectively measured PA. The purpose of this study was to improve the validity of the GPAQ for measuring PA and sedentary behavior by leveraging all information from the self-reported data in a prediction model trained on accelerometer measures in a random sample of mainly white and Latino adults, and to further test this calibration in an independent cohort of white, African American, and Latino adults.

## Methods

### Participants

Data for this study were collected between November 2015 and May 2016 as a part of baseline data collection for Active Ottumwa (AO). AO is a community-based participatory research project designed to increase the PA of all residents of Ottumwa, Iowa through the activation of Lay Health Advisors as Physical Activity Leaders. Ottumwa is a city in southeast Iowa with a population around 25,000, of which 12% are Hispanic or Latino. Ottumwa is largely a processing and manufacturing community [17].

Participants of AO are a random sample of Ottumwa residents (*n* = 139), who were recruited via random digit dialing. The study recruited additional Latino residents via Respondent Driven Sampling [18]. Eligibility requirements included (1) being at least 18 years of age or older; (2) having lived in Ottumwa for at least 6 months; (3) planning to stay in Ottumwa for at least 2 years; and (4) passing physical functioning screening questions modified from NHANES [19].

### Procedures

Individuals who were interested in participation attended a baseline data collection visit at the AO office with a bilingual (English-Spanish) data collector. Participants completed a health survey, and anthropometric measurements. Upon completion of the office visit, participants were fitted with an ActiGraph GT9X Link (ActiGraph LLC, Pensacola, FL) accelerometer, which served as the criterion measure of PA. The ActiGraph GT9X Link is a small (3.5 × 3.5 × 1 cm), lightweight (14 g), and waterproof tri-axial accelerometer. Its primary accelerometer has a dynamic range of ± 8 gravitational units, and was initialized to collect data at a sampling rate of 80 Hz. The accelerometer was attached to the participants’ non-dominant wrist using a hospital band, and was worn continuously for 7 days, including at night. Participants were instructed to remove the accelerometer 8 days following their office visit, and return it via pre-paid mail.

On the accelerometer removal day, a research assistant contacted the participants and administered the GPAQ over the phone. The GPAQ asked participants to recall their PA and sedentary behavior over the past 7 days, to coincide with the time the accelerometer was worn. The GPAQ was designed to fit the specific population being assessed through the use of tailored examples. Within each PA domain, participants were asked to report time spent in moderate-intensity and vigorous-intensity PA. Participants were also queried about sedentary behavior by reporting time spent sitting or reclining. The GPAQ was scored by calculating the average time per day spent in each activity domain and intensity.

### Independent sample validation

The GPAQ calibration models were tested on an independent sample representing a dissimilar population. The validation sample data were collected between December 2014 and September 2016 as a part of baseline data for the Living Well Together (LWT) study in Polk County, Iowa. Participants of the LWT study were low income, urban, obese adults (BMI ≥ 30 kg/m^2^) who were the primary caregiver of a child six to 12 years old, and were drawn from an overall study population that was 29% African American, 25% Latino, and 41% white.

Similar data collection procedures were followed for the LWT study, as for AO. Participants completed baseline data collection and were fitted with an ActiGraph GT9X Link accelerometer on their non-dominant wrist using a hospital band. Participants wore the accelerometer for seven consecutive days, including nights, and were asked to remove it on the eighth day following their baseline data collection visit. A research team member contacted the participants via telephone on the eighth day to complete the GPAQ, where they were queried about their PA over the preceding 7 days.

### Data processing

For both samples, upon receipt of the accelerometer by the research team, data were downloaded and screened for wear-time and monitor malfunction. Participant sleep time was manually extracted using the Physical Activity and Health Outcomes Laboratory sleep algorithm (University of Iowa, Iowa City, IA; details available from corresponding author). Sedentary behavior (min/d) was extracted using the Staudenmayer decision tree algorithm [20] programmed into R software (version 3.3.1). Moderate-, and vigorous-intensity PA (min/d) were calculated using the Hildebrand algorithm [21] in a Visual Basic analysis program developed at the University of Iowa (R. Paulos, University of Iowa, Iowa City, IA). Accelerometry data were matched with the corresponding GPAQ data for analysis. Participants were excluded if they had less than four valid days of accelerometer data collection. A valid day was defined as having at least 10 h of awake data collection.

### Statistical analyses

The analytical goal was to take the information included in the self-reported GPAQ and construct prediction models using each of the outcomes from the accelerometry data as the response variable. To this end, four multiple linear regression models were trained corresponding to sedentary behavior, moderate-intensity PA, vigorous-intensity PA, and MVPA (min/d) as measured by ActiGraph. Covariates for each of the four models included gender, age, and self-reported min/d of sedentary behavior, moderate-intensity PA, vigorous-intensity PA, and the sum of moderate- and vigorous-intensity PA corresponding to the GPAQ scores. Due to the lack of a clear linear relationship between these variables and the ActiGraph data (correlations between ActiGraph and corresponding GPAQ values ranged from 0.04 to 0.19), several transformations of each variable were considered, namely log, square root, linear, and quadratic relationships. Importantly, our purpose here is not to estimate and make inference on the relationship between the GPAQ self-report outcomes and the corresponding outcomes as measured by accelerometry, but rather to take the self-report outcomes and some easy to measure demographic variables and make accurate predictions of the outcomes as would be measured by accelerometry.

In addition to the four covariate transformations mentioned above, break point analysis was also considered. Break point analysis entails estimating from the data a change point in the covariate space where the linear relationship between the response and the covariate changes [22]. That is, at a break point the intercept and the slope for the covariate under consideration changes. This method works by adding a covariate binary indicator which equals one if the variable under consideration is greater than its break point and zero otherwise; the interaction of this binary indicator with the variable itself is also considered. For each continuous covariate and for each of the four response variables, a bivariate break point analysis was performed and the ensuing non-linear relationship was considered to be the fifth possible covariate transformation.

AO data were split into training (80%) and testing (20%) samples. The training sample was used to create the multiple linear regression models. Akaike Information Criterion (AIC) was used to perform model selection on the models created based on the training sample. Specifically, for each of the four responses, an exhaustive search over the 2 × 6^5^( = 15,552) candidate models was performed, and for each model fit the AIC was computed, thus giving a measure of predictive ability for each of the 15,552 models for each of the four outcomes [23]. The model with the best AIC on the training data was selected for each outcome and subsequently evaluated on the out of sample data. Mean Squared Prediction Errors (MSPE) were calculated for the AO testing and LWT independent validation samples. Percent reductions in MSPE were evaluated to measure error relative to the raw GPAQ estimates of sedentary behavior and PA.

In addition to the analysis described above, for comparative purposes we implemented a simple outcome-matching model only utilizing gender, age, and the matching GPAQ outcome, e.g., using gender, age, and self-reported sedentary behavior to predict ActiGraph measured sedentary behavior. All analyses were performed using R software (version 3.3.1). Break point analyses were performed using the strucchange package [24].

## Results

### Participants

The AO sample used in our analysis included 108 participants (*n* = 74 females; *n* = 34 males). Average age of participants was 49.4 years (range: 19.8–68.7 years). Participants wore the ActiGraph accelerometer for an average of 7 days (range: 4–10 days) and had an average awake wear time of 980 min/d (16.3 h). Participants accumulated 72.8 min/d of MVPA on average, measured with the ActiGraph, and reported an average of 104.4 min/d of MVPA on the GPAQ. Model-adjusted GPAQ MVPA was 74.9 min/d. Participants had 543.2 min/d of sedentary behavior measured with the ActiGraph, and reported an average of 338.9 min/d of sedentary behavior on the GPAQ. Model-adjusted GPAQ sedentary behavior was 534.8 min/d. Raw GPAQ scores (min/d) were weakly correlated with ActiGraph measured PA (*r* = 0.04 for moderate-intensity PA; *r* = 0.19 for vigorous-intensity PA) and sedentary behavior (r = 0.19).

The LWT independent validation sample included 120 participants (*n* = 111 females; *n* = 9 males). Average age of participants was 36.5 years (range: 23.4–62.3 years). Participants wore the ActiGraph accelerometer for an average of 7 days (range: 4–10 days) and had an average awake wear time of 984 min/d (16.4 h). Participants accumulated 83.7 min/d of MVPA on average, measured with the ActiGraph, and reported an average of 68.8 min/d of MVPA on the GPAQ. Model-adjusted GPAQ MVPA was 84.8 min/d. Participants had 510.7 min/d of sedentary behavior measured with the ActiGraph, and reported an average of 294.9 min/d of sedentary behavior on the GPAQ. Model-adjusted GPAQ sedentary behavior was 545.3 min/d. Table 1 shows the descriptive statistics of the testing, training, and independent validation samples. Table 2 shows the breakdown of GPAQ reported MVPA by domain. The table demonstrates the vast differences in how each sample accrued daily MVPA. The AO sample accumulated the majority of MVPA from work-related activity (66.4%), while the LWT sample accumulated the majority of MVPA from recreation (50.0%). Additionally, the LWT sample had more MVPA from travel than the AO samples.

**Table 1:** Descriptive Statistics of Active Ottumwa Training and Testing Samples and Living Well Together Validation Sample

	Active Ottumwa Training Sample (*n* = 86) Mean (SD)	Active Ottumwa Testing Sample (*n* = 22) Mean (SD)	Living Well Together Validation Sample (*n* = 120) Mean (SD)
Age (year)	49.1 (14.1)	50.6 (13.7)	36.5 (8.1)
Gender (% female)	67.4	72.7	92.5
Race (%)			
White	81.4	86.4	42.2
Latino	14.0	9.1	23.5
African American	3.5	4.5	23.5
Other	1.1	0.0	10.8
Height (cm)	164.9 (9.20)	165.2 (9.75)	163.4 (10.8)
Weight (kg)	85.5 (23.8)	87.7 (25.8)	108.3 (25.6)
BMI (kg/m^2^)	31.2 (7.8)	32.1 (8.64)	40.8 (11.3)
ActiGraph moderate-intensity PA (min/d)	74.0 (61.8)	60.8 (46.0)	82.0 (56.6)
GPAQ moderate-intensity PA (min/d)	72.8 (95.5)	91.9 (193.9)	47.2 (77.0)
ActiGraph vigorous-intensity PA (min/d)	0.97 (2.13)	3.29 (11.7)	1.69 (5.69)
GPAQ vigorous-intensity PA (min/d)	23.9 (47.9)	42.8 (85.9)	21.6 (44.7)
ActiGraph sedentary behavior (min/d)	535.1 (117.8)	574.6 (154.4)	510.7 (211.1)
GPAQ sedentary behavior (min/d)	332.7 (208.0)	363.2 (210.4)	294.9 (201.0)

*SD* = standard deviation; *cm* = centimeters; *kg* = kilograms; *m* = meters; *PA* = physical activity; *min/d* = minutes per day; *GPAQ* = Global Physical Activity Questionnaire

**Table 2:** Percent of Reported Moderate- and Vigorous-Intensity Physical Activity Spent in Each Activity Domain

	Active Ottumwa Training Sample (*n* = 86)	Active Ottumwa Testing Sample (n = 22)	Living Well Together Validation Sample (*n* = 120)
Work (%MVPA)	63.89	78.17	36.76
Travel (%MVPA)	5.45	1.65	13.21
Recreation (%MVPA)	30.66	20.18	50.03

%MVPA – percentage of total reported daily moderate- and vigorous-intensity physical activity

### Calibration

**Fig. 1:**
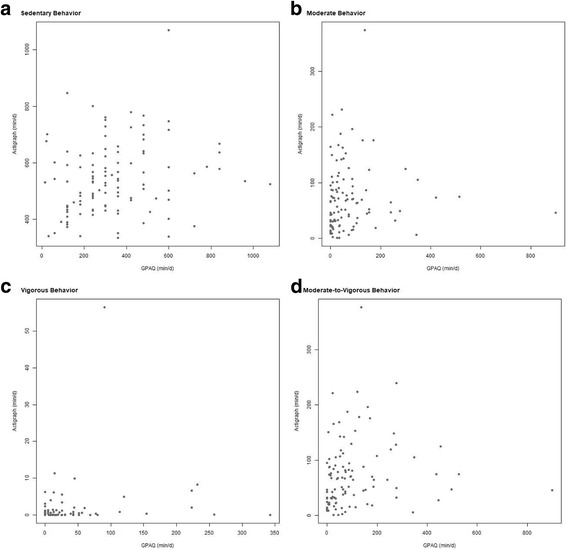
Global Physical Activity Questionnaire Reported and ActiGraph Measured Sedentary Behavior and Physical Activity. Raw GPAQ reported sedentary behavior and PA (min/d) plotted against ActiGraph measured sedentary behavior and PA (min/d)

We developed multiple linear regression models to the AO training set utilizing as covariates gender, age, GPAQ reported sedentary behavior, GPAQ reported moderate-intensity PA, GPAQ reported vigorous-intensity PA, and GPAQ reported MVPA to predict accelerometer measured sedentary behavior and PA. Figure 1 shows the scatterplots of the self-reported GPAQ outcomes and the corresponding accelerometry outcome. This figure shows that there is a weak association between the self-report and accelerometry data. Table 3 corroborates this by giving the proportion of variance explained (*R*^2^) for the outcome-matching model using only gender, age, and the matching GPAQ outcome variable as covariates, as well as that for the final calibration model chosen by AIC. The proposed calibration model shows considerable improvement in fit over the simpler outcome-matching model. Table 4 shows the model parameters for final calibration models selected by AIC. Note that the break factor for a particular variable corresponds to a change in the intercept of the regression model when that variable takes values larger than the break factor cutoff. Similarly, the interaction of a variable with its break factor corresponds to a change in the slope term when that variable exceeds the cutoff value. For example, in the model predicting moderate-intensity PA, if the sum of a subject’s self-reported moderate- and vigorous-intensity PA (GPAQ MVPA) is less than or equal to 113.6, then the intercept of the prediction model is 348.2 and the slope corresponding to GPAQ MVPA is − 0.07, otherwise the intercept is 563.1 (= 348.2 + 214.9) and the slope for GPAQ MVPA is − 1.22 (= − 0.07–1.15).

**Table 3:** Proportion of Variance Explained for the Outcome-Matching Models and the Proposed Calibration Models

Outcome	***R***^**2**^ for Outcome-Matching Model	***R***^**2**^ for Final Calibration Model
Sedentary behavior	0.025	0.097
Moderate-intensity PA	0.144	0.341
Vigorous-intensity PA	0.177	0.364
MVPA	0.156	0.282

*PA* = physical activity; *MVPA* = moderate- and vigorous-intensity physical activity

**Table 4:** Multiple Linear Regression Prediction Models for ActiGraph Measured Sedentary Behavior and Physical Activity

Model Parameters	Estimate	Break Factor Cutoff
Sedentary Behavior		
Intercept	616.5	
GPAQ Moderate-Intensity PA	−0.03	
GPAQ Moderate-Intensity PA Break Factor	− 78.0	0.00
GPAQ Vigorous-Intensity PA	−0.45	
Moderate-Intensity PA		
Intercept	348.2	
SQRT GPAQ Sedentary Behavior	−1.87	
GPAQ Moderate-Intensity PA^2^	1.65 × 10^−3^	
GPAQ Vigorous-Intensity PA^2^	3.54 × 10^− 3^	
LOG Age	−65.3	
GPAQ MVPA	−0.07	
GPAQ MVPA Break Factor	214.9	113.6
Interaction: (GPAQ MVPA)*(MVPA Break Factor)	−1.15	
Vigorous-Intensity PA		
Intercept	10.5	
GPAQ Moderate-Intensity PA^2^	2.91 × 10^−5^	
GPAQ Vigorous-intensity PA^2^	7.84 × 10^−5^	
Age	−0.29	
Age Break Factor	−9.77	27.6
GPAQ MVPA	−0.01	
GPAQ MVPA Break Factor	2.14	38.6
Interaction: (Age)*(Age Break Factor)	0.28	
Interaction: (GPAQ MVPA)*(MVPA Break Factor)	−6.55 × 10^−3^	
MVPA		
Intercept	540.8	
LOG GPAQ Sedentary Behavior	−12.8	
Age	−15.0	
Age Break Factor	− 356.0	29.1
LOG GPAQ MVPA	5.78	
Interaction: (Age)*(Age Break Factor)	13.7	

*GPAQ* = Global Physical Activity Questionnaire; *PA* = physical activity; *SQRT* = square root; *MVPA* = moderate- and vigorous-intensity physical activity; * = interaction

### Validation

Predictive ability of the multiple linear regression models was assessed using the percent reduction of Mean Squared Prediction Errors (MSPE) from the model-adjusted GPAQ values versus the raw GPAQ values. The percent reductions in MSPE are shown in Table 5, indicating that the calibrated GPAQ values led to dramatic reductions in error. From the table we see that the MSPE was reduced by 77.7% when using the model-adjusted GPAQ values versus the raw GPAQ values for sedentary behavior in the AO validation sample. In the same sample, error was reduced by 66.4% for moderate-intensity PA, 98.3% for vigorous-intensity PA, and 94.2% for MVPA. Similarly, MSPE reductions ranged from 61.3% to 98.6% in the LWT independent validation sample.

**Table 5:** Percent reduction in Mean Squared Prediction Errors for Active Ottumwa and Living Well Together Validation Samples From Proposed Calibration Models

Predicted Variable	Active Ottumwa % Reduction in MSPE	Living Well Together % Reduction in MSPE
Sedentary behavior (min/d)	77.7%	80.6%
Moderate-intensity physical activity (min/d)	66.4%	61.3%
Vigorous-intensity physical activity (min/d)	98.3%	98.6%
Moderate- and vigorous-intensity physical activity (min/d)	94.2%	77.5%

*MSPE* = Mean Squared Prediction Errors; *min/d* = minutes per day

**Fig. 2:**
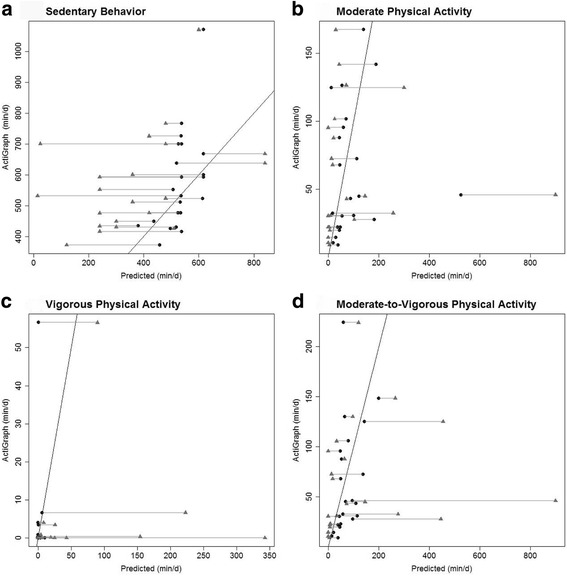
Global Physical Activity Questionnaire Reported, Model-Adjusted, and ActiGraph Measured Sedentary Behavior and Physical Activity. Triangles = raw GPAQ reported sedentary behavior and PA (min/d) plotted against ActiGraph measured sedentary behavior and PA (min/d) Circles = model-adjusted sedentary behavior and PA (min/d) plotted against ActiGraph measured sedentary behavior and PA (min/d). Self-reported raw GPAQ values are connected to their corresponding model-adjusted GPAQ value with a horizontal line. Covariates include sedentary behavior, moderate-intensity PA, vigorous-intensity PA, MVPA, and age.

**Fig. 3:**
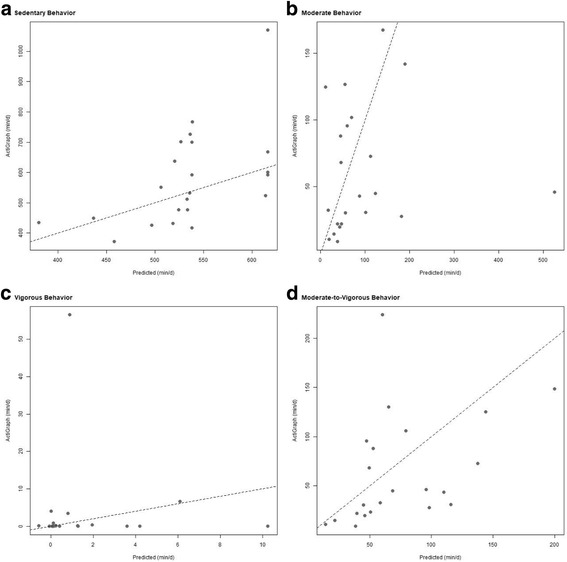
Model-Adjusted and ActiGraph Measured Sedentary Behavior and Physical Activity. Model-adjusted sedentary behavior and PA (min/d) plotted against ActiGraph measured sedentary behavior and PA (min/d)

Figure 2 shows the improvement in predictive value of the GPAQ using the multiple linear regression models detailed in Table 4. The vertical axis represents ActiGraph measured (A) sedentary behavior; (B) moderate-intensity PA; (C) vigorous-intensity PA; and (D) MVPA in (min/d), and the horizontal axis represents the predicted values. The triangles represent self-reported raw GPAQ values (min/d) of each participant in the validation sample, while the circles represent the model-adjusted GPAQ values (min/d). Self-reported raw GPAQ values are connected to their corresponding model-adjusted GPAQ value with a horizontal line. This figure indicates that the adjusted GPAQ values pull the raw GPAQ values closer to the truth (i.e., closer to the *y* = *x* line), thus showing the vast improvement in predictive accuracy of sedentary behavior and PA when using the multiple linear regression models over the raw GPAQ scores. Figure 3 shows these same plots but without the GPAQ values included. Despite the dramatic reduction in error, the predictions are still rather noisy. For the model predicting sedentary behavior, there seems to be an issue with obtaining relatively few unique predicted values; these two things imply that further work in this area would be beneficial.

## Discussion

Calibrating PA questionnaires to objective measures of PA is a useful way to denoise this self-reported data. Additionally, this method can be utilized to compare self-reported PA data with objective measures, as well as better understand how these two measures can be used together. The current study demonstrates the feasibility of this approach by calibrating the GPAQ to 7 days of ActiGraph data.

The multiple linear regression models dramatically reduced prediction errors in both the AO testing sample and the LWT independent validation sample. Raw GPAQ scores overestimated MVPA by 31.6 min/d, and underestimated sedentary behavior by 204.3 min/d in the AO sample using the ActiGraph as ground truth. While the model-adjusted mean GPAQ scores were within 2.1 min/d and 8.4 min/d of the ActiGraph measured mean MVPA and sedentary behavior, respectively. It is important to note that these reductions in error were seen in out-of-sample predictions, meaning they were applied to separate samples than what the models were built on. Additionally, using an independent validation approach we were able to show the model’s utility in a *dissimilar* population, who underreported PA rather than over-reported it, as the AO population did. The two populations also accumulated PA in differing domains; while the AO sample accumulated 66.4% of their MVPA from work-related activity, the LWT sample only accumulated 36.8% from work. The majority of the LWT sample’s MVPA came from recreational activity (50.0%), and they also accumulated substantially more travel-related MVPA than the AO sample (13.2% for LWT versus 4.8% for AO). The LWT population has lower income and education levels than the AO population, and they are 42.4% White, 23.5% African American, and 23.5% Hispanic or Latino, while the AO population is 82.4% White, 3.7% African American, and 13.0% Hispanic or Latino. This provides some assurance of the calibration model’s accuracy for new studies on dissimilar populations.

Adding age and performing data transformations on the self-reported GPAQ values improved PA prediction. Models utilized only data readily available from the questionnaire to improve usability in other studies where more thorough examinations, including anthropometric and objective PA data, are not available. The potential application of this calibration model includes improved predictions of PA in large sample surveillance data, longitudinal studies, and pre−/post-measurements where objective measures of PA are not feasible.

Strengths of the current study include the use of a population-wide random sample, as well as the use of data splitting to validate the models. Additionally, a major strength of the study lies in the ability to evaluate the predictive ability of our models on an independent sample from a disparate population with differing racial and socio-demographic make-up. The study utilizes one of the most commonly used PA questionnaires in current practice, which increases its usability for other researchers. Limitations of the study include a mostly white sample, while an effort was made to oversample the Latino population in Ottumwa. The study sample is also mostly female, particularly in the independent validation sample. Finally, the training data set was necessarily small, potentially limiting the range of PA observed to be smaller than that which might be observed in a larger study.

## Conclusions

The logistics related to large scale public health research make self-reported PA data an attractive option. This manuscript demonstrates a unique way to calibrate and compare self-reported questionnaire data to objective PA measures to improve accuracy of PA estimates, as well as provides a better understanding of the disconnect between the two. Importantly, error in MVPA estimates was reduced by up to 94.2%. Additionally, it helps researchers to harmonize different measures of the same variable, and to understand how self-reported and objective measures can be used together to create a more cohesive PA measure. Ultimately, improved PA measurement will lead to better understanding of PA determinants, as well as the complex relationships between PA and health outcomes, and to assess intervention effectiveness. Researchers can utilize this calibration model for self-reported GPAQ data via a free Shiny web application [25].

## Abbreviations

AIC: Akaike Information Criterion
AO: Active Ottumwa
cm: Centimeter
GPAQ: Global Physical Activity Questionnaire
kg: Kilogram
LWT: Living Well Together
m: Meter
min/d: Minutes per day
MSPE: Mean Squared Prediction Error
MVPA: Moderate- and vigorous-intensity physical activity
PA: Physical activity
PAR: Physical activity recall
SD: Standard deviation
SQRT: Square root
WHO: World Health Organization
